# Circulating Endothelial Progenitor Cells and the Risk of Vascular Events after Ischemic Stroke

**DOI:** 10.1371/journal.pone.0124895

**Published:** 2015-04-13

**Authors:** Joan Martí-Fàbregas, Raquel Delgado-Mederos, Javier Crespo, Esther Peña, Rebeca Marín, Elena Jiménez-Xarrié, Ana Fernández-Arcos, Jesús Pérez-Pérez, Alejandro Martínez-Domeño, Pol Camps-Renom, Luís Prats-Sánchez, Francesca Casoni, Lina Badimon

**Affiliations:** 1 Department of Neurology, Hospital de la Santa Creu i Sant Pau, IIB-Sant Pau, Barcelona, Spain; 2 Cardiovascular Research Center, Hospital de la Santa Creu i Sant Pau, IIB-Sant Pau, Barcelona, Spain; Collège de France / CNRS / INSERM, FRANCE

## Abstract

**Background and Purpose:**

We evaluated the hypothesis that the number of circulating EPC could be associated with the risk of stroke recurrence (SR) or vascular events (VE) after an ischemic stroke.

**Methods:**

We studied prospectively consecutive patients with cerebral infarction within the first 48 hours after the onset. We recorded demographic factors, vascular risk factors, previous Rankin scale (RS) score, and etiology. We analyzed EPC counts by flow cytometry in blood collected at day 7 and defined EPC as CD34+/CD133+/KDR+ cells. Mean follow-up was 29.3 ± 16 months. We evaluated SR as well as VE. Patients were classified as to the presence or absence of EPC in the circulation (either EPC+ or EPC-). Bivariate analyses, Kaplan-Meier survival curves and Cox regression models were used.

**Results:**

We included 121 patients (mean age 70.1±12.6 years; 65% were men). The percentage of EPC+ patients was 47.1%. SR occurred in 12 (9.9%) and VE in 18 (14.9%) patients. SR was associated significantly with a worse prior RS score, previous stroke and etiology, but not with EPC count. VE were associated significantly with EPC-, worse prior RS score, previous stroke, high age, peripheral artery disease and etiology. Cox regression model showed that EPC- (HR 7.07, p=0.003), age (HR 1.08, p=0.004) and a worse prior RS score (HR 5.8, p=0.004) were associated significantly with an increased risk of VE.

**Conclusions:**

The absence of circulating EPC is not associated with the risk of stroke recurrence, but is associated with an increased risk of future vascular events.

## Introduction

Circulating endothelial progenitor cells (EPC) were described in 1997 by Asahara et al[1]. EPC are immature endothelial circulating cells mobilized from the bone marrow that are released into the bloodstream. These cells have an essential physiologic role in vascular homeostasis: they are necessary to repair the injured endothelium and to enable neovascularization after ischemia[2–6].

Several studies have demonstrated that EPC counts are inversely related to the number of traditional vascular risk factors[3,5–7]. Thus, EPC counts are surrogate markers for the risk of vascular events. Their counts may be an index of the vascular risk of a patient better than any other vascular risk factor. It is likely that low counts of EPC may produce vascular events due to the inability of EPC to perform their physiological role. In agreement with this reasoning, two studies[8,9] in patients with coronary artery disease found that EPC counts predicted the occurrence of cardiovascular events during follow-up.

We could not find similar studies in patients with stroke. So, we conducted a study in patients with recent ischemic stroke. We measured EPC counts at day 7 after the onset of stroke and prospectively evaluated the occurrence of ischemic stroke recurrences and other vascular events during follow-up. Our hypothesis was that low EPC counts are associated with a high risk of cerebrovascular events, and other vascular events.

## Material and Methods

The hospital Ethics Committee (Hospital de la Santa Creu i Sant Pau, Barcelona, Spain) approved the study, and written informed consent was obtained from participating patients or their legal representatives. We studied prospectively consecutive patients who had an acute ischemic stroke. These patients were admitted to the Neurology Department at the Hospital de la Santa Creu i Sant Pau (Barcelona, Spain). All of the patients were included within the first 48 hours after the onset of stroke.

Exclusion criteria were: (1) A previous modified Rankin scale (RS) score higher than 2; (2) A NIHSS score of 0; (3) The lack of processing of the blood sample within 30 minutes after extraction, as this was the pre-defined time window to obtain reliable results. We evaluated the same patients in a previous study[10] in which we searched for markers of EPC counts and for the association between EPC counts and short-term prognosis in the acute stage of ischemic stroke.

### EPC measurement

Blood samples (4-ml) were obtained by venopuncture and collected in EDTA tubes at day 7 after the onset of stroke. We found the highest count found after this period in a preliminary study[10].

We measured EPC counts by flow cytometry. Cells were classified as EPC when they were positive for the following three surface markers: CD34 (a marker of hematopoietic stem cells), CD133 (a marker of immature hematopoietic stem cells) and KDR (a marker of endothelial protein). We refined the method previously described for measuring EPC[11]. In brief, EDTA-blood samples were stained with a phycoerythrincyanin–conjugated anti-CD34 monoclonal antibody (Beckman-Coulter, Marseille, France), phycoerythrin–conjugated anti-CD133 monoclonal antibody (Miltenyi-Biotec, Bergisch-Gladbach, Germany) and carboxyfluorescein–conjugated anti-KDR monoclonal antibody (R&DSystems, Wiesbaden, Germany). Isotype-matched antibodies were used as controls. After staining, the samples were fixed with 0.2% formaldehyde for 2 hours and then analyzed by flow cytometry (EPICS XL, Beckman Coulter, Fullerton, CA, USA). We settled on the appropriate gate for mononuclear cells and used EXPO32 ADC software (Beckman Coulter) to identify triple-positively-stained cells. We expressed our results as the proportion of positive cells for the three markers in relation to the total number of gated cells. Typically, 300.000 total events were acquired to determinate the percentage of the CD34**+**/ CD133**+/**KDR**+** subpopulation in the gate.

### Clinical data

Patients were followed-up every 3 months during a mean follow-up of 29.3 ± 16 months. The occurrence of vascular events was recorded. Most patients were interviewed face to face, but with patients who could not attend the scheduled visits, the required information was obtained by telephone from either the patient or a well-informed relative. A physician expert in stroke collected the data; if a stroke recurrence, a vascular event or death occurred outside of our center, medical records were obtained when possible (examination of hospital records and medical files of the patients’ family doctor). During follow-up, secondary prevention measures followed the guidelines of the Cerebrovascular Group from the Spanish Neurological Society[12,13]. Investigators blinded to the EPC level of the patients did all of the data analyses and event adjudication.

For each patient, we recorded the following data: (1) demographic factors (age and gender); (2) presence at inclusion of traditional vascular risk factors including high blood pressure, diabetes mellitus, hypercholesterolemia, coronary artery disease, smoking habit, alcohol abuse, peripheral artery disease, atrial fibrillation, previous transient ischemic attack, previous cerebral infarct; (3) treatment with any statin at any dose during follow-up, treatment with intravenous rt-PA; (4) stroke etiological subtype, according to the SSS-TOAST classification[14]; (5) NIHSS score and prior RS score at admission; (6) Stroke Recurrence (SR), that was defined as the sudden onset of a focal neurologic deficit in a location consistent with the territory of a major cerebral artery, either the same or different from the index stroke. Most patients had a new acute ischemic lesion detected by neuroimaging. But SR was diagnosed also in patients without follow-up neuroimaging in whom the investigator adjudicated the event as stroke; (7) Vascular Event (VE), that was defined as the diagnosis of any of the following: Ischemic or hemorrhagic stroke, myocardial infarction, aortic dissection, acute peripheral limb ischemia, acute mesenteric ischemia and sudden death; (8) Vascular death (sudden death or death after any VE) and death from any cause.

### Statistical analyses

Results are expressed as percentages for categorical variables and as mean (SD) or median and interquartile range for the continuous variables. Since we found many patients with a complete absence of EPC[10], patients were classified with respect to the presence or absence of EPC in the circulation as either EPC+ or EPC-. Thus, patients with 1 or more EPC were combined in the same EPC+ group. Proportions were compared using the Chi-square or the Fisher test, while continuous variables between groups were compared with the Student’s t-test. We determined bivariate predictors of SR and VE using the Kaplan-Meier’s survival curves with significance evaluated by the log-rank test. Cox regression analysis was used to calculate adjusted hazard ratio (HR) as a measure of relative risk for SR and VE and the association with variables. A multivariable Cox regression model was constructed to include all of the variables with a p≤0.1 in the bivariate analysis and variables with clinical relevance. A p value<0.05 was considered as statistical significance.

## Results

We studied 165 patients at baseline. Due to a final diagnosis other than stroke, 19 patients were excluded. Additionally, 25 patients were excluded at day 7 for the following reasons: death (n = 6), early discharge (n = 4), withdrawal of consent (n = 1), and defective blood sampling (n = 14).

**Fig 1:**
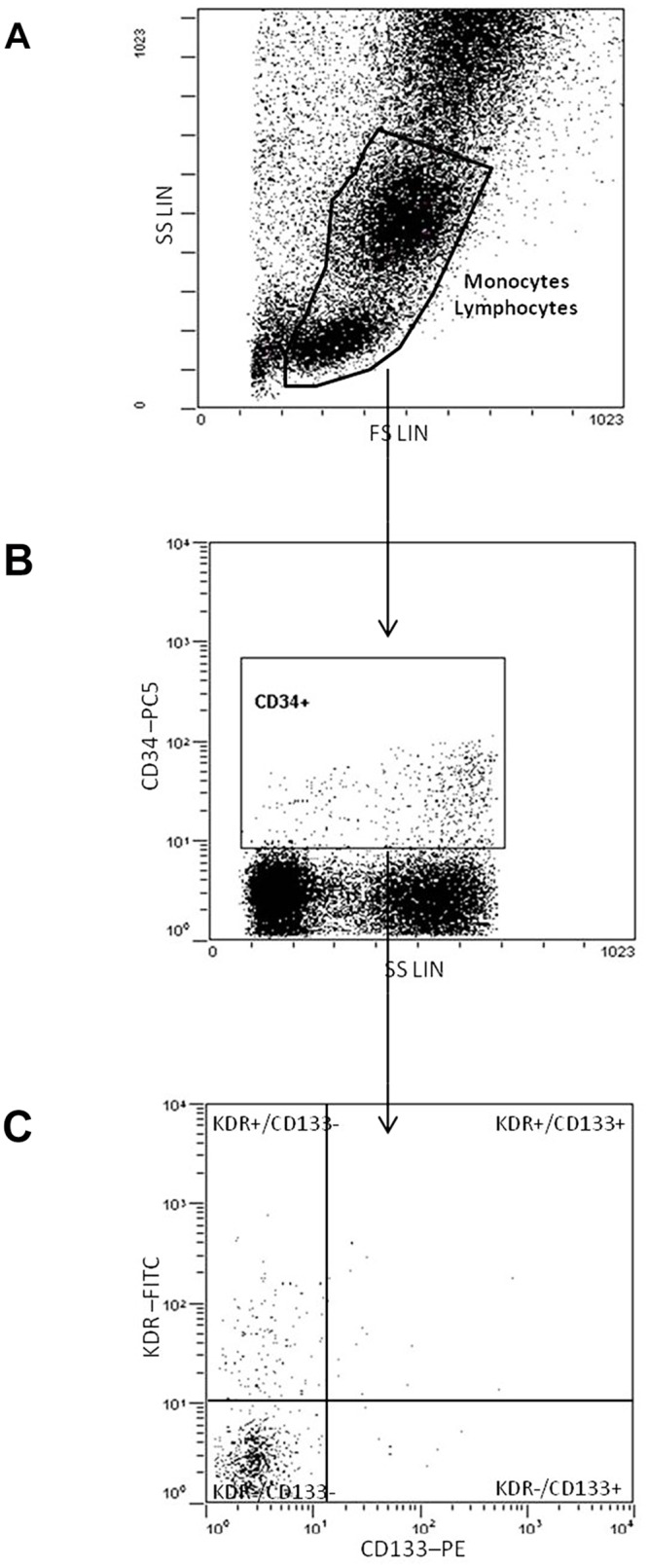
Example of gating strategy of EPCs by FACS. (A) Lymphocytes and monocytes were gated in lineal FSC/SSC plot. (B) CD34+ positive events were gated in a lineal SSC/log CD34-PC5 plot as a subpopulation of lymphocytes and monocytes gate. (C) CD34+/KDR+/CD133+ events within CD34+ gate were detected in a log AC133-PE/log KDR-FITC plot.

Therefore, we studied a total of 121 patients, whose mean age was 70.2 ± 12.6 years (range 36–90), and 79 (65%) of them were men. Table 1 lists the frequency of traditional vascular risk factors, etiological subtypes and severity of the neurological deficit at admission. Overall, EPC counts were seen rarely in the peripheral blood (0.007421 ± 0.137567%). In fact, EPC were detectable in 57 patients (Group EPC+, 47.1%) and not detectable in the remaining 64 patients (Group EPC-, 52.9%). For EPC+ patients the counts of EPC were 0.015754 ± 0.0021841%. In Fig 1 we show an example of the gating strategy for the classification of a cell as EPC. As shown in Table 1, the distribution of variables in patients with and without EPC was equivalent regarding the demographics, vascular risk factors, statin treatment, intravenous thrombolysis, stroke subtype and severity of the neurologic deficit at admission.

**Table 1:** Distribution of Variables in Patients with (EPC+) and Without (EPC-) Circulating EPC.

Variable	All (n = 121)	EPC+ (n = 57)	EPC- (n = 64)	p value
Age, y	70.1 (12.6)	70.9 (12.7)	69.4 (12.6)	0.52
Gender (% men)	65.3%	66.7%	64.1%	0.84
High blood pressure	76%	43.5%	56.5%	0.20
Diabetes Mellitus	28.1%	28.1%	28.1%	1
Hypercholesterolemia	35.5%	35.1%	35.9%	0.53
Coronary Artery Disease	19.8%	22.8%	17.2%	0.29
Smoking	22.3%	19.3%	25%	0.42
Alcohol abuse	5.8%	7%	4.7%	0.70
Peripheral Artery Disease	10.7%	7%	14.1%	0.25
Atrial Fibrillation	23.1%	26.3%	20.3%	0.51
Congestive Heart Failure	24%	26.3%	21.9%	0.67
Previous TIA	11.6%	12.3%	10.9%	1
Previous cerebral infarct	11.6%	12.3%	10.9%	1
Statins prior to admission	30.6%	33.3%	28.1%	0.55
Statins during follow-up	93.8%	93.6%	93.9%	1
Intravenous thrombolysis (rt-PA)	22.3%	28%	17.1%	0.11
Stroke etiological subtype				0.20
Large-artery atherothrombosis	14.9%	8.8%	20.3%	
Cardiac embolism	40.5%	49.1%	32.8%	
Small-vessel disease	4.1%	10.5%	17.2%	
Uncommon etiology	26.4%	5.3%	3.1%	
Cryptogenic	14%	26.3%	26.6%	
Prior Rankin scale score				0.54
0	89.3%	86%	92.2%	
1	6.6%	5.3%	4.7%	
2	4.1%	8.8%	3.1%	
NIHSS at admission (Median, IQR)	6 (3–14)	6 (3,14)	6(2.25,12)	0.96
Vascular Event	18 (14.9%)	6 (10.5%)	12 (18.8%)	0.058
Ischemic stroke recurrence	12 (9.9%)	4 (7%)	8 (12.5%)	0.104

After a mean follow-up of 29.3 ± 16 months SR occurred in 12 (9.9%) patients and VE in 18 (14.9%) patients. SR was classified as transient Ischemic attack in 2, intracerebral hemorrhage in 1 and ischemic stroke in 9 patients. VE consisted of 12 SR, 4 patients with acute myocardial infarction, 1 acute lower limb ischemia due to embolism from atrial fibrillation and 1 acute mesenteric ischemia. Table 2 presents the clinical characteristics of patients with and without a SR, and with and without a VE.

**Table 2:** Association of Variables in Patients with and Without Stroke Recurrence (SR) or Vascular Event (VE), according to Kaplan-Meier Curves and log-rank Test (Mantel-Cox).

Variable (Kaplan-Meier curve, log-rank (Mantel-Cox))	SR	VE
	p value	p value
Age, y	**0.029**	**0.001**
Gender (% men)	0.84	0.81
Previous Rankin scale score	**0.001**	**<0.0001**
High blood pressure	0.94	0.78
Diabetes Mellitus	0.56	0.66
Hypercholesterolemia	0.65	0.97
Coronary Artery Disease	0.53	0.088
Smoking	**0.035**	0.12
Alcohol abuse	0.92	0.66
Peripheral Artery Disease	0.35	**0.005**
Atrial Fibrillation	0.81	0.44
Congestive Heart Failure	**0.033**	0.11
Previous cerebral infarct	**0.011**	**0.027**
Statin during follow-up	0.20	0.29
Stroke etiological subtype	0.14	**0.003**
NIHSS at admission	0.67	0.55
EPC+	0.104	0.058

**Fig 2:**
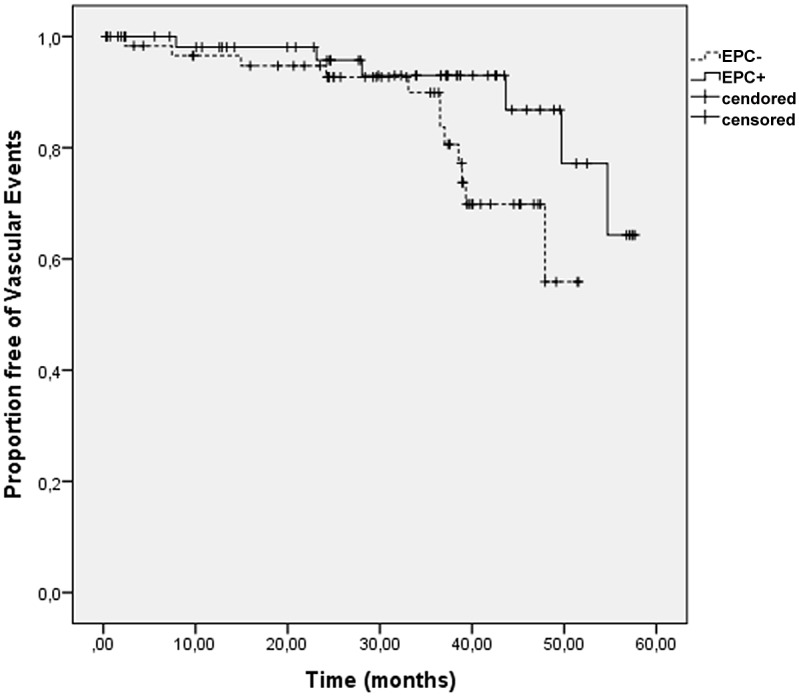
Kaplan-Meier Survival Curves for Vascular Events in Patients with (EPC+) and Without (EPC-) Circulating EPC. Result of the Log-rank test Comparing Both Groups, p = 0.058.

Patients suffering from a VE during follow-up were older (p = 0.001), had a worse previous RS score (p<0.0001), a higher frequency of peripheral artery disease (p = 0.005), previous ischemic stroke (p = 0.027) and large atherosclerosis or undetermined as etiologic subtype (p = 0.003) when compared with their counterparts without VE. In the VE group there was a higher frequency of EPC- patients, although the difference with patients without VE was borderline (p = 0.058, Fig 2). The Cox regression model for VE showed that EPC- (HR 7.07, 95%CI 1.94–25.7, p = 0.003), age (HR 1.08, 95%CI 1.02–1.14, p = 0.004) and a worse prior RS score (HR 5.8, 95% CI 1.73–19.75, p = 0.004) were significantly associated with an increased risk for VE during follow-up.

**Fig 3:**
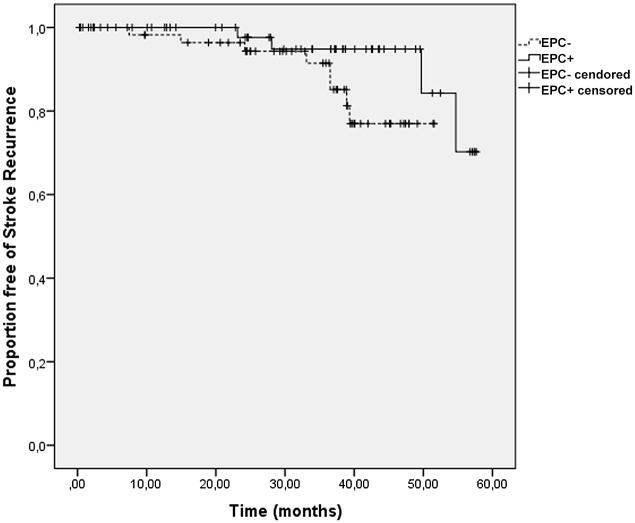
Kaplan-Meier Survival Curves for Stroke Recurrence in Patients with (EPC+) and Without (EPC-) Circulating EPC. Result of the Log-rank test Comparing Both Groups, p = 0.104.

It is interesting that compared to patients without SR, patients with a SR during follow-up were older (p = 0.029), had a worse previous RS score (p = 0.001), and a higher frequency of smoking (p = 0.035), congestive cardiac failure (p = 0.033) and previous ischemic stroke (p = 0.011). Although the SR group had a high frequency of EPC- patients, the difference with patients without SR was not significant (p = 0.104, Fig 3).

Likewise, EPC counts were not associated with mortality. During follow-up 25 patients died (21%), 13 from the EPC- group and 12 from the EPC+ group (p = 0.82). Likewise, deaths from vascular causes were equal in both groups. Vascular death occurred in 9 patients (7.4%), 4 from the EPC- group and 5 from the EPC+ group (p = 0.73). The cause of death was unknown for 9 patients (5 from the EPC- group and 4 from the EPC+ group). In the remaining patients the cause of death was not related to vascular etiologies.

## Discussion

We evaluated the association between circulating EPC counts and vascular outcomes after a follow-up of patients with ischemic stroke. We demonstrated that a complete absence of circulating EPC predicts future vascular events in patients with acute ischemic stroke. The risk for VE was 7-fold higher in the EPC- group compared with the EPC+ group. The risk of a VE was associated also to high age and worse previous RS score. Interestingly, the predictive value of the EPC count was independent from these well-known prognostic variables. Moreover, we found a higher frequency of ischemic stroke recurrences in patients with no circulating EPC as compared to those with circulating EPC. However, this association was not statistically significant. Notably, EPC counts were not associated with mortality.

The counts of EPC are low in patients with traditional vascular risk factors, but increase with exercise and in patients receiving statins. Therefore, the EPC count can be viewed as an estimate of the individual vascular risk. In addition, it has a higher predictive value than any single risk factor[3,5–7,15]. We found a complete absence of circulating EPC in about half of our patients. Also, previous studies reported the scarcity of these cells in peripheral blood[16].

Two previous studies reported the predictive value of the level of EPC and demonstrated its clinical relevance in patients with ischemic heart disease. One study[9] included 43 healthy subjects, 44 patients with stable ischemic heart disease and 33 with acute coronary syndromes. After a median follow-up of 10 months they found that lower levels of EPC (as measured by flow cytometry) independently predicted a cardiovascular event with a hazard ratio of 3.9. Another study[8] included 519 patients with stable ischemic heart disease and analyzed the levels of circulating EPC by flow cytometry. After a follow-up of 12 months, they found that low levels of EPC were associated with cardiovascular events and death from cardiovascular causes. To our knowledge there are no studies that investigated whether the predictive value of EPC levels is applicable for patients with ischemic stroke. We believe that our results are compatible with the results of these studies.

Most EPC are generated in the bone marrow and are released into the blood to aid in the repair of damaged blood vessel endothelium. However, circulating EPC provide only a limited pool of cells and their level results from the dynamic balance between production and consumption[5]. A low number of EPC may result from an insufficient production by the bone marrow, an impaired mobilization or an excessive consumption. The data are sparse and inconsistent regarding estimates of EPC counts and its correlation with relevant variables in the acute stage of stroke[10]. Several studies reported that ischemic stroke triggers the production and mobilization of EPC[17–19], and their level peaks at day 7 after stroke and decreases thereafter[2,10,17,19]. Also, higher EPC counts at day 7 (but not at admission) are related to a better neurologic outcome through different potential mechanisms[10,17,19]. Therefore, we chose this time point to evaluate the value of the EPC counts in assessing the risk of recurrences. According to our findings, a patient with a sufficient production of EPC shortly after an ischemic stroke may be protected against a VE during follow-up. In contrast, a patient with insufficient production of EPC may have a high risk of a VE. However, our study was not designed to evaluate whether a sample obtained in the chronic stage would be predictive also. It is likely that an increase or decrease of EPC counts during follow-up may have also prognostic value.

The mechanism by which a high EPC count protects (or a lower EPC count increases the risk) of a VE is not clear, but it is consistent with the role played by these cells in regulating the homeostasis of the vascular system. According to several studies, the structure and function of endothelium is impaired in patients with ischemic stroke[20]. Thus, a low count or absence of EPC may not be able to maintain its function to repair the injured endothelium, thus favoring an ischemic event[16]. Endothelial dysfunction is thought to be associated more strongly with lacunar and large-artery atherothrombosis subtypes than with cardioembolism[17,21–23]. Some studies have reported also the endothelial impairment in patients with cardioembolic stroke due to atrial fibrillation[24] and others found that endothelium dysfunction is independent of the stroke subtype[20,25]. We found that the counts of EPC were not related to the etiologic subtype. This suggests that the absence of circulating EPC is a risk factor for a VE irrespectively of the etiology of the ischemic stroke. As endothelium dysfunction is also involved in coronary artery disease and atherosclerosis in other territories[26–28], it is not surprising that in our study EPC counts were associated with VE other than ischemic stroke. In addition to endothelial repair, the protection offered by EPC may also be related to its ability to increase collaterals, as demonstrated in coronary artery disease[29], and its role in the maintenance of perfusion and cerebral metabolism[30]. Important limitations of our study are the relative small number of patients evaluated, a relative small number of VE and the absence of a control group without stroke. This limits the strength of conclusions that can be drawn from this study. Since most VE were ischemic stroke recurrences, it is likely that a larger sample of patients would have shown a significant association between reduced or absence of EPC and increased risk of stroke recurrence. A larger sample and longer follow-up would provide enough power to clarify the importance of stroke subtypes. Moreover, the cause of death was unknown for 9 patients and it is possible that it was vascular for some of them, which could have changed our results. Furthermore, during follow-up, although patients were treated according to national guidelines, we did not evaluate the adherence and compliance of patients to the secondary prevention measures, a factor that is related clearly to the risk of recurrence. Finally, EPC are part of a heterogeneous cell population and also there is no consensus on their definition[2,15,31]. We obtained our counts according to the usually accepted procedure, although we evaluated only by a quantitative test and did not by a functional test such as colony-forming properties. However the same molecular pathways regulate quantitative and functional phenomena[3].

To our knowledge this study is the first to measure the relationship between EPC count and the risk of future vascular events in patients with acute ischemic stroke. Patients with the absence of EPC had a higher frequency of VE. If a larger and independent cohort confirms these findings, the monitoring of circulating EPC may become a surrogate marker of the risk of a VE. Moreover, our results suggest that providing EPC to a patient might have therapeutic value by stopping or slowing the progression of vascular disease[15,16].
